# Temperature Dependence of Optical Properties of MoS_2_ and WS_2_ Heterostructures Assessed by Spectroscopic Ellipsometry

**DOI:** 10.3390/nano15010076

**Published:** 2025-01-06

**Authors:** Hoang Tung Nguyen, Van Long Le, Thi Mai Nguyen, Xuan Khuyen Bui, Thi Giang Nguyen, Nhat Linh Nguyen, Xuan Au Nguyen, Tae Jung Kim

**Affiliations:** 1 Institute of Materials Science, Vietnam Academy of Science and Technology, Hanoi 100000, Vietnam; 2 Department of Physics, Kyung Hee University, Seoul 02447, Republic of Korea; xuanau@khu.ac.kr

**Keywords:** MoS_2_ and WS_2_ heterostructures, spectroscopic ellipsometry, dielectric function, temperature dependence, critical point, exciton

## Abstract

We report the complex dielectric function *ε* = *ε*_1_ + *iε*_2_ of MoS_2_/WS_2_ and WS_2_/MoS_2_ heterostructures and their constituent monolayers MoS_2_ and WS_2_ for an energy range from 1.5 to 6.0 eV and temperatures from 39 to 300 K. Comparisons between the optical properties of the heterostructures and their monolayers were conducted. Critical-point (CP) energies of the heterostructures were traced back to their origins in the monolayers. Low-temperature measurements confirmed the existence of only three excitonic CPs from 1.5 to 2.5 eV due to the overlap of trion *B*^−^ of the MoS_2_ monolayer and exciton *A*^0^ of the WS_2_ monolayer. Due to the dielectric screening effect, most CPs exhibit red shifts in the heterostructures compared to their monolayer counterparts.

## 1. Introduction

Recently, transition-metal dichalcogenides (TMDCs) have emerged as a new class of semiconductors due to their unique low-dimensional properties. These materials, often referred to as “beyond-graphene”, display remarkable physical and chemical characteristics, including transitions from indirect to direct band gaps, quantum yield enhancement, and alteration of spin–valley coupling by changing from multilayers to monolayers. The surge in TMDC research is also driven by the possibility of stacking different monolayers to make heterostructures, allowing for access to notable properties due to interlayer coupling [1,2], interlayer charge transfer [3,4], and Moire patterns [5,6].

Among various TMDC materials, MoS_2_ stands out as the pioneering and most extensively studied member, showcasing standard TMDC traits such as a van der Waals layered structure and an indirect-to-direct band gap transition in monolayers. WS_2_ has also attracted significant interest for its high quantum yield in 2D systems and substantial spin–orbit coupling characteristic of heavy metals. The vertical stacking of MoS_2_ and WS_2_ monolayers to form heterostructures creates highly desirable properties in optoelectronic fields, particularly in light detection and light-harvesting devices. Recent advancements in fabrication techniques have enabled the production of high-quality and uniform MoS_2_ and WS_2_ monolayers and their heterostructures, bringing these materials closer to commercial applications. To fully leverage the potential of TMDC materials and their heterostructures, a comprehensive understanding of light–matter interaction described through the dielectric function of these 2D systems is essential [7]. Critical points (CPs) can be studied by analyzing dielectric functions. Knowledge of the CP energies of 2D TMDC systems, both monolayers and heterostructures, is essential for band gap engineering, allowing for access to the intrinsic properties of these materials, including their electronic structures and exciton binding energies. Despite the extensive research on monolayer MoS_2_ and WS_2_ over the past decade, only a few works can be found to report on the dielectric function and CP energies of these monolayers [8,9,10,11,12,13], and even fewer studies on the heterostructures [14,15].

Spectroscopic ellipsometry (SE) is renowned for being a powerful and non-destructive tool for accurately studying the intrinsic optical properties of two-dimensional van der Waals materials, allowing for the simultaneous acquisition of both the real and imaginary parts of the dielectric functions [16]. The dielectric function of 2D heterostructures obtained by SE represents their actual dielectric response, which is significantly influenced by interlayer coupling and the screening effect, as opposed to pseudo-dielectric functions for complex surfaces [14,17]. Furthermore, investigating the optical properties of 2D TMDC systems by SE at cryogenic temperatures could reveal the intrinsic characteristics of these materials by significantly reducing thermal noise, as demonstrated in previous works [13,18,19,20]. In a recent work, X. Zhu et al. successfully reported the dielectric functions and CPs of WS_2_/MoS_2_ heterostructure. However, their study was limited to room temperature and data on the reversed form of the heterostructure are still lacking. Despite significant advancements, a systematic study on TMDC heterostructures at low temperatures is not yet available as far as we know. Such research is crucial for uncovering new insights into the optical behavior of 2D materials in heterostructure forms, thereby advancing their applications in nanoelectronics and photonics.

This work presents the dielectric function and CP energies of monolayer MoS_2_, monolayer WS_2_, and their heterostructures MoS_2_/WS_2_ and WS_2_/MoS_2_ within a 1.5–6.0 eV spectral range. The samples were measured at low temperatures of up to 300 K, with the lowest measured temperatures being 70 K and 39 K for the monolayers and the heterostructures, respectively. The monolayers were fabricated using the chemical vapor deposition method by 2D Semiconductor Inc. (Scottsdale, AZ, USA) and were then transferred onto each other to form heterostructures. The quality of these structures was confirmed through Raman, Photoluminescence (PL), and SE techniques. The intrinsic dielectric properties of the monolayers and heterostructures were extracted from pseudo-dielectric functions measured using point-by-point fitting analysis with the appropriate thicknesses. CP energy values were identified via standard line shape analysis from derivative spectra. As temperature decreased, we observed blue shifts and sharpening of the CPs. Due to the reduction in thermal noise at low temperatures, we found new CPs of the heterostructures that were not discernible at room temperature. Importantly, exciton and trion properties are well resolved via their temperature dependences. At low temperatures, the *A* and *B* exciton structures of the monolayers were identified as trions (*A*^−^, *B*^−^) for monolayer MoS_2_ and neutral excitons (*A*^0^, *B*^0^) for monolayer WS_2_ in an energy range from 1.5 to 2.6 eV. However, only three peaks (denoted as *X*_1_, *X*_2_, and *X*_3_) could be observed in the heterostructure in the same energy range. By conducting a systematic temperature dependence study, we were able to determine that the origins of the excitons and the other CPs in the heterostructures were traced back to the constituent monolayers. Most other CPs in the monolayers are found in the heterostructures, except for CP *E*_II_ of the WS_2_ monolayer. CP energies tend to red shift in comparison to their monolayer origins. However, trion *A*^−^ of monolayer MoS_2_ exhibits a blue shift when MoS_2_ is under WS_2_ in the heterostructure and a red shift when MoS_2_ is on top of WS_2_.

## 2. Materials and Methods

### 2.1. Sample Fabrication and Characterization

**Figure 1:**
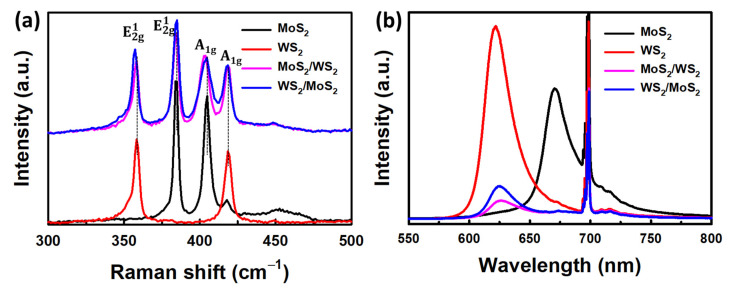
(**a**) Raman and (**b**) PL spectra of MoS_2_ WS_2_ and monolayers and their MoS_2_/WS_2_ and WS_2_/MoS_2_ heterostructures.

Monolayer MoS_2_, monolayer WS_2_, and their heterostructures MoS_2_/WS_2_ and WS_2_/MoS_2_ are commercially available from 2Dsemiconductors Inc. (Scottsdale, AZ, USA), where the MoS_2_ and WS_2_ monolayers were grown on Si substrates covered with SiO_2_ by atmospheric pressure chemical vapor deposition and low-pressure chemical vapor deposition, respectively [21]. The monolayers were then transferred onto a sapphire substrate and onto each other by a wet polymethyl–methacrylate (PMMA) transfer process [22], as confirmed by 2Dsemiconductors Inc., to form the MoS_2_/WS_2_ and WS_2_/MoS_2_ heterostructures. We employed Raman and PL performed with 532 nm laser excitation under ambient conditions to measure the received MoS_2_/WS_2_ heterostructure. The laser was focused to a spot approximately 1 μm in diameter on the sample using a 100× objective lens with a numerical aperture (N.A.) of 0.8, and the scattered light was subsequently collected. Raman scattering measurements were performed with the XploRA PLUS Raman spectroscopy instrument from Horiba in Kyoto, Japan, with a grating groove density of 1800 lines/mm. The PL signal was collected within a wavelength range of 500–800 nm using a QE65 Pro spectrometer (grating density: 900 grooves/mm, Ocean Optics, Delray Beach, FL, USA). The results are shown in Figure 1. In the Raman data shown in Figure 1a, we marked the vibration modes corresponding to each monolayer. It was confirmed that Raman vibration of the heterostructure was present in the region from 300 to 500 cm^−1^, where the vibration modes of the heterostructures match the superposition of the vibration mode of each constituent monolayer [23]. In this work, monolayer MoS_2_ and monolayer WS_2_ display characteristic $E_{2g}^{1}$–A_1g_ modes positioned at 383.9–404.9 cm^−1^ and 356.6–419.0 cm^−1^, respectively. Figure 1b presents the measured PL spectrum of the heterostructures in comparison to those of the MoS_2_ and WS_2_ monolayers. The PL intensity of the heterostructures is well quenched due to typical interlayer charge transfer in type II heterojunctions, as discussed in Ref. [24]. This means the two monolayers are in good contact. It should be noted that, after transferring, the heterostructure was baked in a vacuum condition to enhance the interlayer interaction [23]. Therefore, our MoS_2_ and WS_2_ heterostructures are in good condition for SE experiments.

### 2.2. SE Temperature Dependence

Since Moiré patterns may occur in TMDC junctions [25,26], which results in different interlayer coupling and optical properties, we carefully examined the sample by measuring it at various spots using SE focusing probes (M2000-FI ellipsometer, J. A. Woollam Co., Inc., Lincoln, NE, USA), which reduce the beam spot to a hundred-micrometer scale. Even though SE is highly sensitive to the optical response of the surface, we could hardly find any difference in the measured data at different spots, confirming the uniformity of the sample at a large scale.

The samples were fixed to a cold-finger sample holder using silver paste and enclosed in a cryostat consisting of a stainless-steel high-vacuum chamber, maintaining a base pressure of 10^−8^ Torr evacuated by a turbomolecular pump. This setup minimizes artifacts resulting from condensation at low temperatures. The samples were cooled to below 100 K using a closed-cycle He refrigerator and heated to 300 K with a heating element inside the sample holder. A silicon-diode thermometer was mounted on a dummy sample in the corner of the cold finger to monitor the temperature. Incident light reached the sample through stress-free fused-quartz windows at an angle of 68.2°. Pseudodielectric function data <*ε*> were obtained from 1.5 to 6.0 eV, with temperatures at 20 K intervals from 39 to 150 K and then at 25 K intervals from 175 to 300 K. The ellipsometer is a dual-rotating-compensator configuration (model RC2, J. A. Woollam Co., Inc., Lincoln, NE, USA) at the Multi-dimension Material Convergence Research Center of Kyung Hee University.

## 3. Results and Discussion

### 3.1. Analysis of <ε>

SE determines the pseudo-dielectric function <*ε*> of the sample, including effects from the thin films and the substrate. Therefore, to accurately derive the dielectric functions of the monolayers and their heterostructures, we applied a three-phase model (ambient/monolayer or heterostructure/sapphire substrate) to obtain both the real and imaginary parts of *ε* at each wavelength using a point-by-point fitting approach. In this approach, the thicknesses of the WS_2_ and MoS_2_ monolayers are fixed at 0.80 nm and 0.70 nm, respectively [18,27,28]. The heterostructures are considered single-phase with a thickness of 1.5 nm. It should be noted that the dielectric function and thickness are highly correlated at the monolayer scale. However, fitting with different thicknesses only results in the fluctuation of the dielectric constant values without distorting the line shape or shifting the CP position. Even without incorporating the surface roughness layer in the optical model, the imaginary part of the dielectric function for all samples effectively reached zero below the band gap region. This indicates the high quality of the samples studied in this work and also helps to reduce the number of free parameters needed to describe the data.

**Figure 2:**
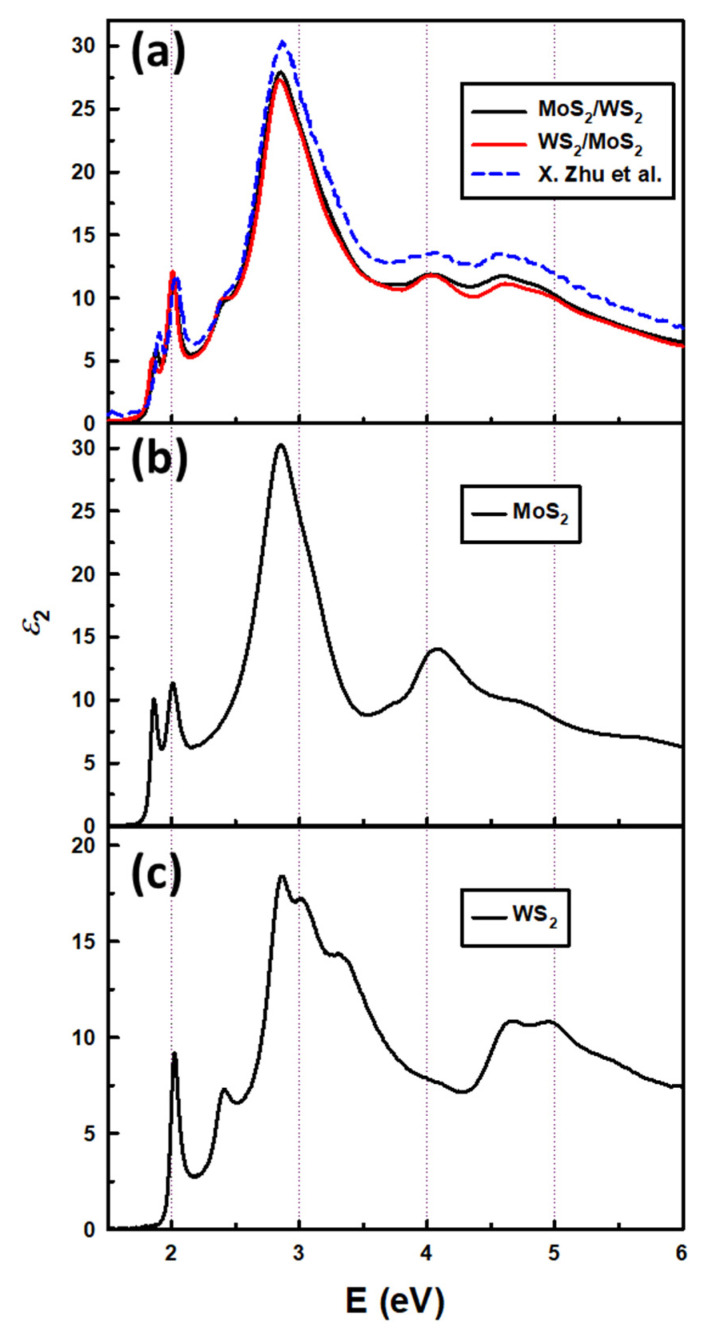
(**a**) Extracted $\varepsilon_{2}$ of the heterostructures in this work at room temperature and data reported by X. Zhu et al. [14] for WS_2_/MoS_2_ and extracted $\varepsilon_{2}$ of (**b**) monolayer MoS_2_ and (**c**) monolayer WS_2_.

In order to confirm the validity of our extracted data, the extracted $\varepsilon_{2}$ of the heterostructures at room temperature was compared with the data reported by X. Zhu et al. [14] as presented in Figure 2a. The imaginary part of the dielectric function of the inclusion monolayers are presented in Figure 2b and Figure 2c for the MoS_2_ and WS_2_ monolayers, respectively. As far as we know, no dielectric function has yet been reported for heterostructures at low temperatures.

**Figure 3:**
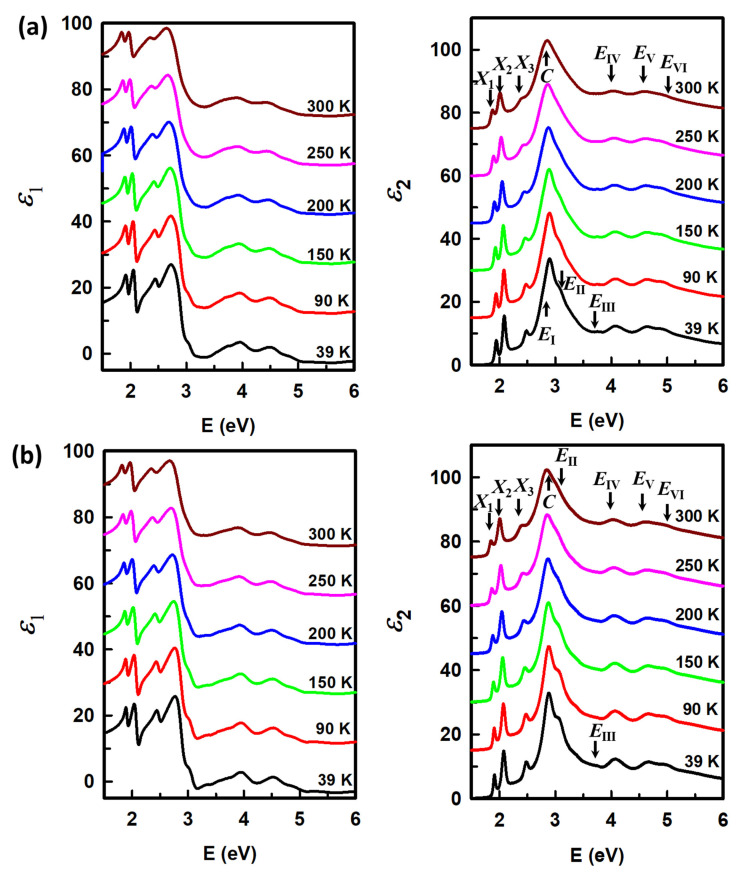
Real and imaginary parts of the dielectric function of (**a**) MoS_2_/WS_2_ and (**b**) WS_2_/MoS_2_ heterostructures from 39 to 300 K. The spectra are offset by an increment of 15 for clarity.

Using the same analysis method at 300 K, we present the real and imaginary parts of the dielectric function of the MoS_2_/WS_2_ and WS_2_/MoS_2_ heterostructures at various temperatures from 39 to 300 K as shown in Figure 3a and Figure 3b, respectively. The spectra are offset by an increment of 15 and the number of temperatures is reduced for clarity. Data for the dielectric functions of the MoS_2_ and WS_2_ monolayers can be found in Ref. [9]. By lowering the temperatures, we observe that the CP structures are blue-shifted and enhanced, which facilitates the detection of new small CPs by avoiding the strong thermal noise at room temperature. These changes are attributed to a significant reduction in electron–phonon interaction and a decrease in lattice constant at low temperatures. The emergence of some CPs at low temperatures, i.e., *E*_I_, *E*_II_, and *E*_III_ in MoS_2_/WS_2_ and *E*_III_ in the WS_2_/MoS_2_ heterostructure, can be pointed out. We are also interested in the *A* and *B* exciton and trion region of the monolayers from 1.5 to 2.6 eV. Since each monolayer contains a pair of *A* and *B* excitons in this region (trions *A*^−^ and *B*^−^ for the MoS_2_ monolayer and neutral excitons *A*^0^ and *B*^0^ for the WS_2_ monolayer, as presented in Ref. [9]), it is expected to observe four excitonic peaks in this region from the dielectric function of the heterostructures. However, one can only observe three CPs denoted as *X*_1_, *X*_2_, and *X*_3_ in Figure 3, even in the spectra measured at 39 K.

**Figure 4:**
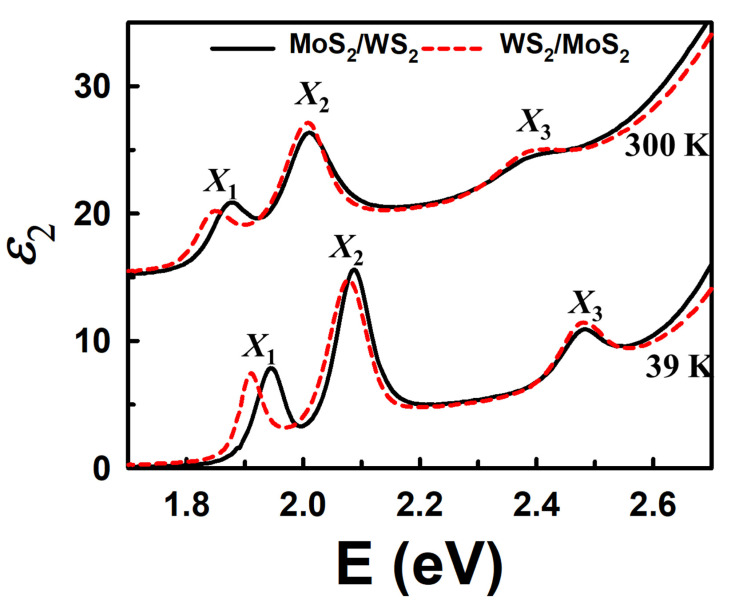
Imaginary parts of the dielectric function of MoS_2_/WS_2_ and WS_2_/MoS_2_ heterostructures at 39 and 300 K on expanded scale around regions of *X*_1_, *X*_2_, and *X*_3_ peaks.

By expanding the region from 1.7 to 2.7 eV in Figure 4, it is confirmed that there are exactly three exciton peaks consistently from 300 K to 39 K. The separation cannot be realized, even though peak structure sharpening and blue shift can be observed. Notably, while CPs *X*_2_ and *X*_3_ of the heterostructures are well matched, *X*_1_ exhibits a significant red shift from the MoS_2_/WS_2_ to the WS_2_/MoS_2_ structure. Since the CP structures are significantly asymmetric due to contributions of transitions from various parts of the Brillouin zone, the CP energies cannot be determined solely by inspection of the original spectra. Therefore, we employed a standard procedure of analyzing second derivatives of the data to determine CP energies at different temperatures.

### 3.2. Critical-Point Analysis

In order to explain the phenomenon of peak reduction from four excitons combined in two monolayers to only three in the heterostructures in the energy region from 1.7 to 2.6 eV, a precise determination of CP position for comparison is required. To differentiate overlapped CP features and to determine exact CP energies, second derivatives $\frac{d^{2}\varepsilon }{dE^{2}}$ were numerically calculated with a Savitzky–Golay algorithm (Table IV of Ref. [29]) to decrease noise and minimize spectra distortions. Regression analysis was performed with the standard analytic CP expression (Equation (1)). $\frac{d^{2}\varepsilon_{1}}{dE^{2}}$ and $\frac{d^{2}\varepsilon_{2}}{dE^{2}}$ were simultaneously fit to determine the parameters better. The best representations of all CPs were yielded on an excitonic line shape (n=−1) and were the same as the cases of each monolayer separately.

(1) $$\frac{d^{2}\varepsilon }{d\omega^{2}}=\left\{\begin{array}{c} n(n-1)Ae^{i\varphi }(\hbar \omega -E+i\Gamma {)}^{n-2},\;n\neq 0\\ Ae^{i\varphi }(\hbar \omega -E+i\Gamma {)}^{-2},\;\;\;\;\;\;\;\;\;\;\;\;\;\;\;\;\;\;\;n=0 \end{array}\right.$$

Here, the CP is described by the amplitude *A*_amp_, threshold energy *E*, broadening *Γ*, and phase Φ as adjustable parameters. The exponent *n* has the values −1, −1/2, 0, and +1/2 for excitonic, 1, 2 (logarithmic), and 3D CPs, respectively. We simultaneously fit real and imaginary parts with all the CPs represented with the excitonic line shape (*n* = −1).

**Figure 5:**
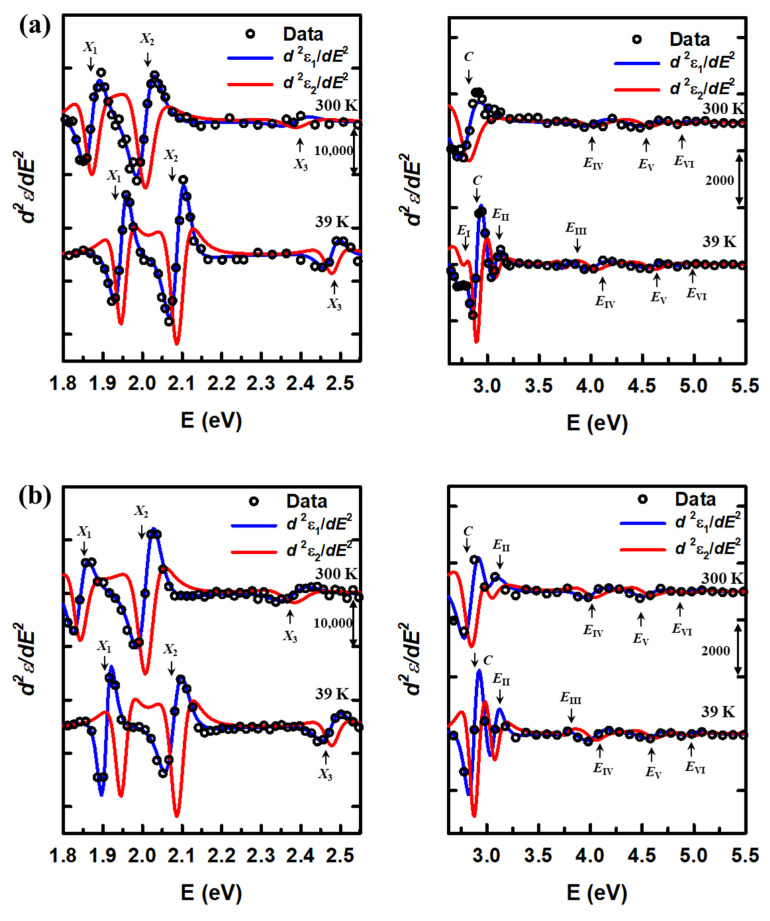
The best fit for $\frac{d^{2}\varepsilon_{1}}{dE^{2}}\;$(blue line) and $\frac{d^{2}\varepsilon_{2}}{dE^{2}}$ (red line) from 1.8 to 2.6 eV and from 2.6 to 5.5 eV for heterostructures (**a**) MoS_2_/WS_2_ and (**b**) WS_2_/MoS_2_ at 39 and 300 K.

The second derivative data of $\varepsilon_{1}$ (open circles) with the best-fit results for $\frac{d^{2}\varepsilon_{1}}{dE^{2}}$ (blue lines) and $\frac{d^{2}\varepsilon_{2}}{dE^{2}}$ (red lines) at 39 and 300 K are shown in Figure 5a and Figure 5b for the MoS_2_/WS_2_ and WS_2_/MoS_2_ heterostructures, respectively. The $\frac{d^{2}\varepsilon_{2}}{dE^{2}}$ data are not shown and a few $\frac{d^{2}\varepsilon_{1}}{dE^{2}}$ data are concealed for clarity. Due to the significant differences in the magnitude of the second derivatives of the CP structures, we divided our analysis into two ranges: 1.8–2.6 eV and 2.6–6.0 eV. The amplitudes of second-derivative spectra in each range are relatively similar, improving fitting accuracy. In the first part from 1.8 to 2.6 eV, only three peaks can be observed in both MoS_2_/WS_2_ and WS_2_/MoS_2_ heterostructures, denoted as *X*_1_, *X*_2_, and *X*_3_. Even at the lowest measured temperature of 39 K, the structures exhibit blue shifts and sharpening due to the reduction in electron–phonon interaction; no separation can be observed. In the second part, from 2.6 to 5.5 eV, the blue shift of CP energies is also observed as temperature decreases. At 39 K, the CP structures are significantly sharper than those at higher temperatures, revealing the existence of new CPs: *E*_I_, *E*_II_, and *E*_III_ in the case of MoS_2_/WS_2_ and *E*_III_ in the case of WS_2_/MoS_2_.

The positions of the CPs at 39 and 300 K for the heterostructures and at 70 and 300 K for the monolayers are listed in Table 1 in detail. The CP energies of the exciton and trion are all listed in order, while the other CPs match their origin from each monolayer. Exciton *X*_1_ of the heterostructures likely originates from *A*^−^ of MoS_2_. *X*_2_ seems to be the superposition of *B*^−^ of MoS_2_ and *A*^0^ of WS_2_. This observation is in good agreement with X. Zhu et al., who also found a single exciton at 2.03 eV (denoted as CP C in the reference). This observation suggests the possibility that *X*_2_ is a single CP combination of the heterostructures or simply a superposition of exciton *A*^−^ of monolayer MoS_2_ and exciton *B*^0^ of monolayer WS_2_. While the *X* CPs are located near the position of their corresponding exciton and trion from the monolayers, exciton *C* of the heterostructure appears at a slightly lower energy than those of the monolayer and is not separated. The existence of a single exciton *C* in both heterostructures is not similar to the CP *X*_2_ combined from the *A* and *B* excitons. This is due to the *A* and *B* excitons being transitions at *K* and *K*’ points of the Brillouin zone, which are strongly confined within each monolayer, while the *C* exciton at *Γ* point has a mixed character, as shown in Ref. [30], i.e., the existence of a single exciton *C* has been theoretically predicted. The analysis of CP energies also finds *E*_I_ in the MoS_2_/WS_2_ heterostructure at temperatures under 200 K. The CP energy of *E*_I_ is 2.75 eV at 39 K. This CP is not found in either heterostructure WS_2_/MoS_2_ or the monolayers in this work. However, in a previous work [18], the authors found the band gap energy *E*_0_ of monolayer WS_2_ to be 2.48 eV and 2.72 eV at 41 K and 300 K, respectively, as listed in Figure 1. This energy aligns well with the *E*_I_ CP observed in the MoS_2_/WS_2_ heterostructure. The origin of this CP is explained as the band gap of the WS_2_ monolayer.

### 3.3. Temperature Dependence of Critical Point Energies

**Figure 6:**
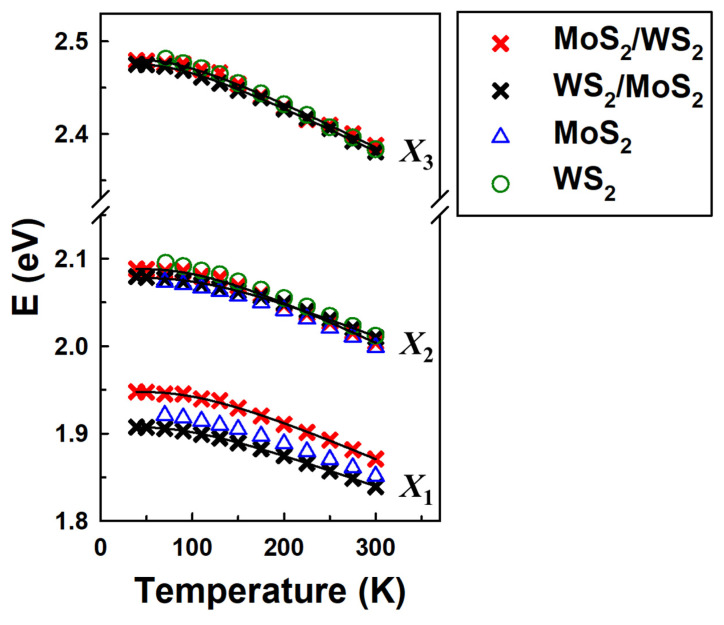
Temperature dependence of the CP energies (✕ symbols) and the best fit (solid lines) for CPs *X*_1_, *X*_2_, and *X*_3_ of the heterostructures. Blue triangles and green circles are trions (*A*^−^, *B*^−^) of monolayer MoS_2_ and neutral excitons (*A*^0^, *B*^0^) of monolayer WS_2_.

**Figure 7:**
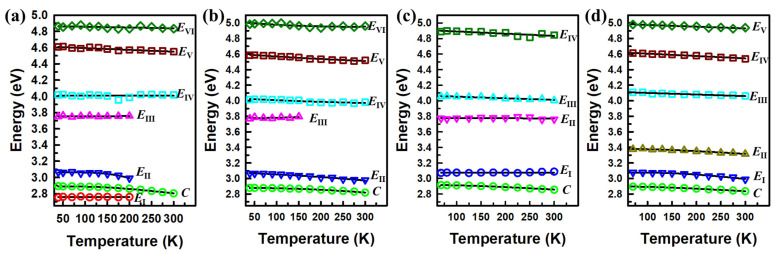
Temperature dependence of CP energies (open symbols) and the best fit (solid lines) for excitons of (**a**) MoS_2_/WS_2_ and (**b**) WS_2_/MoS_2_ heterostructures and their (**c**) MoS_2_ and (**d**) WS_2_ monolayers.

Figure 6 and Figure 7 show the temperature dependence of the CP energies within energy ranges of 1.8–2.6 eV and 2.6–5.0 eV, respectively. The open dots are the CP energies resulting from the second-derivative analysis. The CP data of *X*_1_, *X*_2_, *X*_3_, *E*_I_, C, and *E*_II_ are fit with a phenomenological expression that contains the Bose–Einstein statistical factor for phonons [19,31]:

(2) $$E(T)\;=\;E_{B}-a_{B}\left[1+\frac{2}{e^{\Theta /T}-1}\right]$$

where Θ is the mean frequency of the phonons involved and *E*_B_ is the strength of the interaction between electrons and phonons. *E*_B_, Θ, and *a*_B_ are adjustable parameters. For other CPs which have negligible curvature in temperature dependence, a fit with a linear equation [19,31] was applied:

(3) $$E(T)\;=\;E_{L}-\lambda T$$

where *λ* is the temperature coefficient *−dE/d*T and adjustable parameter *E*_L_. The best fit parameters are listed in Table 2.

In Figure 6, we compare the CPs *X*_1_, *X*_2_, and *X*_3_ of the heterostructures with their potential origins in excitons and trions from each monolayer. The energy position of each CP of the heterostructures and their monolayers has been identified, and their temperature dependence has been determined. The CPs *X*_1_, *X*_2_, and *X*_3_ of the heterostructures MoS_2_/WS_2_ and WS_2_/MoS_2_ are displayed as red and blue ✕ symbols, respectively. Each CP among *X*_1_, *X*_2_, and *X*_3_ of both heterostructures is well fitted to the phenomenological expression that contains the Bose–Einstein statistical factor for phonons; the fitting parameters are listed in Table 2 and Table 3 for the MoS_2_/WS_2_ and WS_2_/MoS_2_ heterostructures, respectively. The temperature dependence of the excitons of the MoS_2_ and WS_2_ monolayers are reported in Ref. [9]. In this work, Figure 6 displays only the energies of trions (*A*^−^, *B*^−^) for monolayer MoS_2_ and neutral excitons (*A*^0^, *B*^0^) for monolayer WS_2_ as blue triangles and green circles, respectively, without fitting lines for clarification. We observed that each CP among *X*_1_, *X*_2_, and *X*_3_ of the heterostructures has a corresponding trion and exciton from the MoS_2_ and WS_2_ monolayers. In detail, *X*_3_ is likely to originate from the *B*^0^ exciton of the WS_2_ monolayer; the temperature dependence of *X*_3_ of both heterostructures is almost identical to exciton *B*^0^. The CP energies of *X*_2_ from both heterostructures are positioned in the middle of *B*^−^ of the MoS_2_ monolayer and *A*_0_ of the WS_2_ monolayer. We suggest *X*_2_ might result from the superposition of these excitons when it comes to the heterostructures. Interestingly, *X*_1_ does not follow the tendency of *X*_3_, even though it also originates from the single trion *A*^−^ of the MoS_2_ monolayer. In comparison to the energy position of trion *A*^−^, *X*_1_ of the MoS_2_/WS_2_ and WS_2_/MoS_2_ heterostructure are clearly blue shifted and red shifted, respectively. This phenomenon suggests that the MoS_2_ monolayer can be more sensitive to the dielectric screening effect than the WS_2_ monolayer. Our observation is in line with data reported in Ref. [14] for the MoS_2_ and WS_2_ monolayers and the WS_2_/MoS_2_ heterostructure, where the authors also observed the red shift of exciton *A* of the MoS_2_ monolayer when it comes to the WS_2_/MoS_2_ heterostructure. Here, we complemented the work with the measurement of the MoS_2_/WS_2_ heterostructure and observed the opposite behavior of the *A* exciton when the MoS_2_ monolayer changed its position in the heterostructure.

Figure 7 shows the CP energies (open dots) above 2.7 eV and their temperature dependences (solid lines), which were fit using a phenomenological expression (Equation (1)) for the *C* and *E*_II_ CPs and a linear equation (Equation (2)) for the *E*_I_ and *E*_III–VI_ CPs. In this figure, the CPs of the heterostructures and their potential originating CPs from the monolayers are presented in the same color. The origins of the CPs in the Brillouin zone of the monolayers are reported in Ref. [10] and Ref. [18] for the MoS_2_ and WS_2_ monolayers, respectively. Almost all the CPs that appear in the monolayers can be found in the heterostructures, except for *E*_II_ of the WS_2_ monolayer. This is in good agreement with the data of X. Zhu et al. [14], who also did not find a CP within the energy of 3.2~3.6 in the heterostructure. In comparison, in the previous work, low-temperature measurements revealed an extra CP *E*_III_ in both heterostructures at temperatures under 200 K, with CP energies closely related to the *E*_II_ of the MoS_2_ monolayer, as shown in Figure 7. In addition, the existence of new CPs, *E*_I_ and *E*_II_, in the MoS_2_/WS_2_ heterostructure is also revealed. Even though a corresponding CP for the *E*_I_ of the MoS_2_/WS_2_ heterostructure was observed from the monolayers in this work, the electronic band gap *E*_0_ of the WS_2_ monolayer was found within the energy range of 2.72 to 2.79 eV in our previous work [18], suggesting the origin of the mentioned CP. The CPs of the heterostructures in this work were also slightly red shifted in comparison to their corresponding CPs from the monolayers due to increased dielectric screening, as discussed by X. Zhu et al. [14].

## 4. Conclusions

We report the spectra of the dielectric function *ε* of MoS_2_ and WS_2_ monolayers and their combined heterostructures, MoS_2_/WS_2_ and WS_2_/MoS_2_, obtained by SE at energies from 1.5 to 6.0 eV and temperatures from under 70 K to 300 K. The energies of the seven CPs, *X*_1_, *X*_2_, *X*_3_, *E*_II_, *E*_III_, *E*_IV_, *E*_V_, and *E*_VI_, were obtained in the WS_2_/MoS_2_ heterostructure and one more CP, *E*_I_, was additionally discovered in the MoS_2_/WS_2_ heterostructure at a cryogenic temperature from the *ε* spectra and their numerically calculated second derivatives. The CPs exhibited blue shifts and enhanced structures upon lowering the temperature due to reduced lattice constants and electron–phonon interactions. The temperature dependence of each CP was fitted to a linear equation or a phenomenological expression with the Bose–Einstein statistical factor. A comparison between the CPs obtained from the heterostructures and the constituent monolayers was performed at various temperatures in the measured energy range to determine the potential origin of the CPs in the heterostructures. At all the measured temperatures, we observed only three peaks of *X*_1_, *X*_2_, and *X*_3_ formed from a combination of two trions and two excitons of the constituent monolayers. The temperature dependence study identified these results as the superposition of trion *B*^−^ of monolayer MoS_2_ and neutral exciton *A*^0^ of monolayer WS_2_ to form the CP X_2_ of the heterostructures. CP *X*_1_, originating from trion *A*^−^ of monolayer MoS_2_, exhibited a red shift and blue shift in the MoS_2_/WS_2_ and WS_2_/MoS_2_ structures, respectively, while the CP *X*_3_, originating from exciton *B*^0^ of monolayer WS_2_, and other CPs at the energies higher than 2.7 eV only slightly red shifted in comparison to the original CPs from the monolayers as a result of an increasing dielectric screening effect. These results provide more profound insights that enable the precise engineering of optoelectronic components and devices based on MoS_2_ and WS_2_ monolayers and their heterostructures.

## Figures and Tables

**Table 1:** CP energies of the heterostructures at 39 and 300 K and the corresponding CPs of the monolayers.

CP Energies (eV)	MoS_2_/WS_2_		WS_2_/MoS_2_		CP Energies (eV)	MoS_2_		CP Energies (eV)	WS_2_	
	39 K	300 K	39 K	300 K		70 K	300 K		70 K	300 K
*X*_1_	1.95	1.87	1.91	1.84	*A* ^−^	1.92	1.85	*A* ^−^	-	-
*X*_2_	2.09	2.00	2.08	2.01	*A* ^0^	-	-	*A* ^0^	2.10	2.01
					*B* ^−^	2.08	2.00	*B* ^−^	-	-
*X*_3_	2.48	2.39	2.48	2.38	*B* ^0^	-	-	*B* ^0^	2.48	2.38
*E*_I_	2.75	-	-	-	-	-	-	*E*_0_ *	2.79 *	2.72 *
*C*	2.89	2.80	2.88	2.82	*C*	2.91	2.86	*C*	2.90	2.84
*E*_II_	3.06	-	3.07	2.98	*E*_I_	3.07	3.09	*E*_I_	3.08	2.98
								*E*_II_	3.38	3.25
*E*_III_	3.75	-	3.77	-	*E*_II_	3.77	3.76			
*E*_IV_	4.02	4.02	4.01	4.01	*E*_III_	4.05	4.00	*E*_III_	4.11	4.06
*E*_V_	4.61	4.55	4.58	4.52				*E*_IV_	4.61	4.54
*E*_VI_	4.86	4.83	4.98	4.96	*E*_IV_	4.89	4.84	*E*_V_	4.98	4.94

* Data from Ref. [18].

**Table 2:** The best-fitting parameters of the temperature dependences of the CPs of the MoS_2_/WS_2_ heterostructure.

Exciton	*E*_B_ (eV)	*a*_B_ (meV)	Θ (K)	*E*_L_ (eV)	*λ* (10^−4^ eVK^−1^)
*X*_1_	2.03	84	347	-	-
*X*_2_	2.18	84	352	-	-
*X*_3_	2.55	70	273	-	-
*E*_I_	-	-	-	2.76	0.06
*C*	3.11	218	545	-	-
*E*_II_	5.09	202	814	-	-
*E*_III_	-	-	-	3.75	0.20
*E*_IV_	-	-	-	4.01	0.01
*E*_V_	-	-	-	4.62	2.23
*E*_VI_	-	-	-	4.87	0.95

**Table 3:** The best-fitting parameters of the temperature dependences of the CPs of the WS_2_/MoS_2_ heterostructure.

Exciton	*E*_B_ (eV)	*a*_B_ (meV)	Θ (K)	*E*_L_ (eV)	*λ* (10^−4^ eVK^−1^)
*X*_1_	1.97	62	312	-	-
*X*_2_	2.16	82	371	-	-
*X*_3_	2.54	68	267	-	-
*C*	2.98	106	463	-	-
*E*_II_	3.11	51	225	-	-
*E*_III_	-	-	-	3.77	1.09
*E*_IV_	-	-	-	4.03	1.99
*E*_V_	-	-	-	4.60	3.11
*E*_VI_	-	-	-	5.00	1.86

## Data Availability

Data are contained within the article.
